# Sandwich Electrochemical Immunosensor for Early Detection of Tuberculosis Based on Graphene/Polyaniline-Modified Screen-Printed Gold Electrode

**DOI:** 10.3390/s18113926

**Published:** 2018-11-14

**Authors:** Umi Zulaikha Mohd Azmi, Nor Azah Yusof, Norzila Kusnin, Jaafar Abdullah, Siti Suraiya, Poh Shing Ong, Nurul Hanun Ahmad Raston, Siti Fatimah Abd Rahman, Mohamad Faris Mohamad Fathil

**Affiliations:** 1 Institute of Advanced Technology, Universiti Putra Malaysia, Serdang 43400, Selangor, Malaysia; umizulaikha.ika@gmail.com (U.Z.M.A.); norzilakusnin87@gmail.com (N.K.); jafar@upm.edu.my (J.A.); 2 Department of Chemistry, Faculty of Science, Universiti Putra Malaysia, Serdang 43400, Selangor, Malaysia; 3 School of Medical Sciences, Universiti Sains Malaysia, Kubang Kerian, Kelantan 16150, Malaysia; ssuraiya@usm.my; 4 NanoMalaysia Berhad, a CLG under the Ministry of Energy, Science, Technology, Environment and Climate Change (MESTECC), Kuala Lumpur 50450, Malaysia; pohshing.ong@nanomalaysia.com.my; 5 School of Biosciences and Biotechnology, Faculty of Science and Technology, Universiti Kebangsaan Malaysia, UKM Bangi 43600, Selangor, Malaysia; nurulhanun@ukm.edu.my; 6 Institute of Nano Electronic Engineering, Universiti Malaysia Perlis, Kangar 01000, Perlis, Malaysia; mohamadfaris@unimap.edu.my

**Keywords:** tuberculosis, electrochemical sensor, screen-printed gold electrode (SPGE), graphene, polyaniline

## Abstract

A rapid and sensitive sandwich electrochemical immunosensor was developed based on the fabrication of the graphene/polyaniline (GP/PANI) nanocomposite onto screen-printed gold electrode (SPGE) for detection of tuberculosis biomarker 10-kDa culture filtrate protein (CFP10). The prepared GP/PANI nanocomposite was characterized using Fourier transform infrared spectroscopy (FTIR) and field emission scanning electron microscopy (FESEM). The chemical bonding and morphology of GP/PANI-modified SPGE were studied by Raman spectroscopy and FESEM coupled with energy dispersive X-ray spectroscopy, respectively. From both studies, it clearly showed that GP/PANI was successfully coated onto SPGE through drop cast technique. Cyclic voltammetry was used to study the electrochemical properties of the modified electrode. The effective surface area for GP/PANI-modified SPGE was enhanced about five times compared with bare SPGE. Differential pulse voltammetry was used to detect the CFP10 antigen. The GP/PANI-modified SPGE that was fortified with sandwich type immunosensor exhibited a wide linear range (20–100 ng/mL) with a low detection limit of 15 ng/mL. This proposed electrochemical immunosensor is sensitive, low sample volume, rapid and disposable, which is suitable for tuberculosis detection in real samples.

## 1. Introduction

Globally, one of the most critical infectious diseases is tuberculosis (TB), which can be fatal [1]. *Mycobacterium tuberculosis (M. tuberculosis)* is the causative agent of TB which can be transmitted via minute aerosol droplets such as coughing, sneezing, or even talking by the infected TB person [2]. This airborne contagious disease poses a particular infection-control challenge since the healthy person can be infected by inhalation of an infectious droplet containing *M. tuberculosis* that suspended in the air. As a result, each year millions of people are infected, making TB the second leading cause of death after the human immunodeficiency virus (HIV) infection [3].

Instead of lung infection, one of the hallmarks of the *M. tuberculosis* is the ability of the bacteria to spread to other parts of the body through the blood circulation in the infected person. In this regard, the consequences of delayed diagnosis of TB are serious for both the prognosis of the patient and onward transmission to drive the epidemic. A diagnostic test that can be conducted at a single-visit, with theoretical 100% accuracy could save 625,000 lives a year if widely implemented [4]. However, even a test with a sensitivity of only 85% and specificity of 97% could save 392,000 lives, equal to 22% of the current annual global deaths attributable to TB [4]. In developing countries, sputum smear microscopy currently remains a main tool of diagnosing TB [5]. However, this method depends on both the quality and bacterial load of the sputum specimen as well as the skills of laboratory technicians. In addition, the smear microscopy is insensitive with the reported sensitivity only about 60% [6]. The culture-based systems have been developed and offer relative improvements in sensitivity over conventional techniques by combining *M. tuberculosis* culture and monitoring in one tube, which known as mycobacteria growth indicator tube (MGIT) [7]. However, this technique is more expensive than microscopy, requires up to ten days culturing time and a high standard of technical competence. Thus, there is an unmet need for reliable diagnostic methods to identify TB disease rapidly and accurately in an economic way, as replacement for the time consuming, complex and costly technique.

These requirements can be accomplished by using biosensors platform, with particular reference to the electrochemical immunosensors. Their low cost, small size and highly sensitive nature are making them have a great impact on the development of rapid assays for TB diagnosis in clinical use. In addition, the use of screen-printed electrodes (SPEs)-based biosensors represents the most favorable strategy for on-site and one-shot sensors (disposable) analysis, since the screen-printed technology has the ability to be easily mass produced [8,9,10]. Moreover, the modification of electrode with nanomaterials will certainly provide an effective way to improve the analytical performance. Graphene (GP)-based nanomaterials have attracted enormous interest in electrochemical sensors due to their extraordinary properties such as large surface area, high electrical conductivity and biocompatibility [11,12,13]. This strategy may be effective in the combination of use with polyaniline (PANI), one of the promising and unique conducting polymers for sensor development [14]. PANI provides fast electron dynamics and excellent electrochemical activity [15]. The conjunction of PANI with GP (PANI/GP) may prevent the introduction of defects into GP substrate and preserves GP’s electrical characteristics.

Genosensor constructed for the detection of nucleic acid hybridization could represent a valid method for TB diagnosis [16,17,18]. Although same-day diagnosis is possible with the molecular assay, usage is currently limited by the relatively high cost. To overcome this challenge, an alternative strategy to develop an economical TB diagnosis test for resource-constrained area is based on the detection of antigens in body fluids. Various tests have been developed to detect Mycobacterial antigens in sputum [19,20], cerebrospinal fluid [21] and urine [22] in TB patients using ELISA for antigen recognition. Indeed, Reither et al. reported that although the ELISA assays were effective in TB detection, however, the assay is semi-quantitative and the sensitivity could be significantly improved [23].

Here, we demonstrate a novel practical approach to develop a disposable immunoassay-based screen-printed gold electrode (SPGE) for TB detection, employing ESAT-6-like protein esxB (CFP10) as a protein biomarker. Noteworthy, CFP10 which is a 10 kDa secreted antigen from *M. tuberculosis* has been selected as a sensitive platform for the detection of early bovine TB infection [24]. An interesting approach based on a two-step strategy, which included GP/PANI-modified electrode and a sandwich immunoassay format was used in order to amplify the detection signal as well as increase the selectivity of the sensor towards the TB-specific biomarkers. The sandwich-type immunoassay format was developed by immobilizing the capture anti-CFP10 antibodies (CapAb) onto GP/PANI-modified SPGE to capture CFP10 in the sample and iron oxide-gold magnetic nanoparticles (Fe_3_O_4_-Au MNPs) conjugated with primary anti-CFP10 antibodies (Ab) served as a signal probe. We also demonstrated the effectiveness of developed immunoassay for the sensitive and quantitative detection of CFP10 in sputum, collected from human samples of TB infected persons. These studies suggested that the blood test-free method through sputum detection of biomarkers such as CFP10 might be of value in the early detection of TB disease, especially in HIV-positive cases and thus could provide wide potential applications in clinical analysis.

## 2. Materials and Methods

### 2.1. Materials and Reagents

*M. tuberculosis* CFP10 antigen and polyclonal anti-CFP10 antibody were obtained from Cusabio (Houston, TX, USA). Tetramethylammonium hydroxide (TMAOH), tetrachloroauric acid hydrate (HAuCl_4_), bovine serum albumin (BSA), potassium hexacyanoferrate (III), tri-sodium citrate, 2-mercaptoethanol (ME), 12-mercaptododecanoic acid (MDDA), graphene powder, aniline, poly (methylvinylether-alt-maleic acid) (PMVEA), ammonium persulfate (APS) and (3-aminopropyl) triethoxysilane (APTES) were purchased from Sigma-Aldrich (St. Louis, MO, USA). Iron (III) chloride (FeCl_3_) and absolute ethanol were purchased from HmbG Chemicals (Hamburg, Germany). N-hydroxysuccinimide (NHS) and 1-ethyl-3-(3-dimethylaminopropyl)-carbodiimide (EDC) were obtained from Alfa Aeser (Lancaster, KA, UK) and Fluka (Ronkonkoma, NY, USA), respectively. Hydroxylamine hydrochloride (NH_2_OH·HCl), sodium hydroxide (NaOH) and iron (II) sulphate (FeSO_4_) were purchased from R&M Chemicals (Essex, UK). All cyclic voltammetry (CV) measurements were performed in 1.00 mM K_3_Fe(CN)_6_ with 50 mM KCl, while the differential pulse voltammetry (DPV) measurements were carried out in 0.01 M phosphate buffered saline (PBS) with pH 7.4. The 0.5 M of sulphuric acid (Sigma, St. Louis, MO, USA) was used to activate the SPGE before modification. Real samples (sputum) were obtained from Hospital Universiti Sains Malaysia (Kubang Kerian, Kelantan, Malaysia). All chemicals are of the qualitative analytical grade. Deionized (DI) water was used to prepare the aqueous solutions.

### 2.2. Apparatus

Electrochemical measurements were performed using potentiostat Metrohm^@^ µAutolab type III (Eco Chemie, Utrecht, The Netherlands) integrated with screen printed junction cable controlled by NOVA 1.11 software. The SPGE was purchased from DropSens (Oviedo, Spain). The diameter of the disk-shaped working electrode was 4 mm. The working electrode and auxiliary electrode were made of gold, whereas the reference electrode was made of silver. The electrodes were all printed on a ceramic support (L 33 × W 10 × H 0.5 mm). All electrochemical measurements were performed at room temperature. Field emission scanning electron microscopy (FESEM) analysis was carried out using an FEI Nova Nanosem 230 microscope equipped with an energy dispersive X-ray (EDX) system. Raman spectroscopy studies were performed with a WITec Alpha 300R microscope while Fourier transform infrared spectroscopy (FTIR) coupled with attenuated total reflectance analysis were executed using Shimadzu.

### 2.3. Synthesis of Iron Oxide Magnetic Nanoparticles Coated with Gold (Fe_3_O_4_/Au MNPs)

Magnetic nanoparticles composed of Fe_3_O_4_ and gold nanohybrid material (Fe_3_O_4_/Au MNPs), in particular, were synthesized via co-precipitation method according to previous reported methods with slight modification [25,26]. Briefly, 25 mL aqueous solution containing of 0.8 M FeCl_3_, 0.4 M FeSO_4_, 40 mM HCl was added drop wise into 1.5 M NaOH (250 mL) solution under vigorous stirring using a non-magnetic stirrer. Black Fe_3_O_4_ MNPs formed immediately, which were separated by NdFeB magnet and washed three times with DI water. The Fe_3_O_4_ MNPs were centrifuged for 15 min at 9000 rpm. Finally, the Fe_3_O_4_ MNPs were dispersed in DI water and stored at 4 °C for further use. To coat the Fe_3_O_4_ MNPs with gold nanoparticles, 5 mL aliquot of the Fe_3_O_4_ MNPs and 1.5 mL of 0.2 M NH_2_OH·HCl was added into 75 mL of 0.01 M TMAOH aqueous solution. The solution was heated at 80 °C under vigorous stirring. Then, 40 mg of HAuCl_4_ in 40 mL deionized water was added into the mixture drop by drop, followed by 100 mL of 15 mM sodium citrate solution was added drop wise. The color of the mixture changed from black to reddish brown gradually. The mixture was heated for another 1 h and then cooled to room temperature. The formed Fe_3_O_4_/Au MNPs were collected by a magnet, followed by centrifuged twice with deionized water and ethanol. Finally, the Fe_3_O_4_/Au MNPs was dried at 70 °C in a vacuum oven for 1 h.

For carboxylation steps, 2-mercaptoethanol and 12-mercaptododecanoic acid (ME-MDDA) was self-assembled on Fe_3_O_4_/Au MNPs surface by the well-known gold-thiol (Au-S) chemistry. Then, 10 mg of the Fe_3_O_4_/Au MNPs were suspended in 1 mM ethanolic ME-MDDA and incubated for 24 h at room temperature. The carboxylated nanoparticles were washed several times with water and dispersed in 0.01 M PBS solution with pH 7.4.

### 2.4. Immobilization of Primary Anti-CFP10 Antibody on Fe_3_O_4_/Au MNPs

Briefly, 1 mL functionalized Fe_3_O_4_/Au MNPs was mixed with 1 mL 25 µg/mL antibody (Ab) solution followed by incubation for 2 h. Subsequently, Fe_3_O_4_/Au-Ab was collected by a magnet and washed three times with 0.01 M PBS solution. Then, Fe_3_O_4_/Au-Ab was re-dispersed in 1 mL of 1% BSA for 2 h and separated again using a magnet. Finally, Fe_3_O_4_/Au-Ab was washed with PBS again and re-dispersed the particles in PBS solution. The particles were stored at 4 °C for further use.

### 2.5. Preparation of GP/PANI Nanocomposite

The method to prepare GP/PANI nanocomposite was done according to an in-situ polymerazation procedure previously reported by Mohamad and group [18]. Briefly, 0.2 M aniline and 3 wt % PMVEA were dissolved in deionized water. Then, the solution was cooled in a refrigerator at 4 °C for 60 min and followed by adding a pre-cooled aqueous solution of 0.2 M APS. Next, 50% weight ratio of GP to aniline was added into the mixture and placed at room temperature at least 6 h in order to complete the whole reaction for polymerization process. The black-green precipitate was filtered and rinsed with methanol, deionized water and acetone for several times. Finally, the GP/PANI nanocomposite product was dried overnight at 40 °C.

### 2.6. Modification of SPGE-Based on Sandwich Immunoassay Format

**Figure 1:**
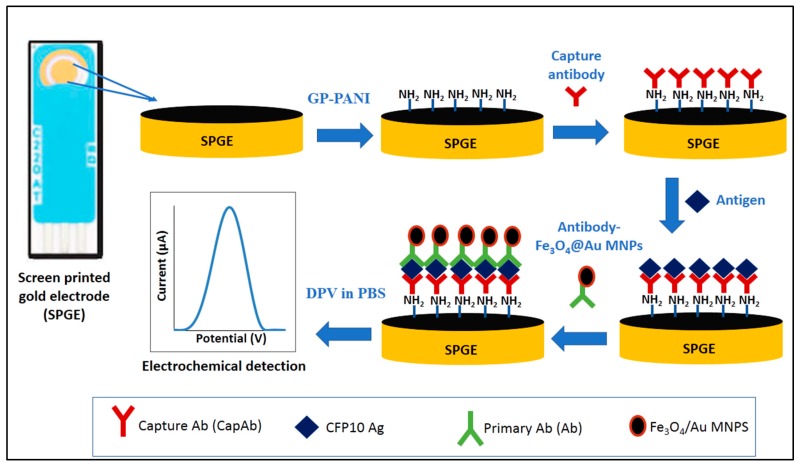
Schematic diagram of the fabrication process of GP/PANI-modified SPGE in sandwich immunoassay format.

The development of sandwich immunosensor for CFP10 detection in this study is depicted in Figure 1. Firstly, the electrode was activated with 0.5 M H_2_SO_4_ solution using CV between 0.0–1.6 V for 40 cycles, scan rate of 100 mV/s and dried at room temperature. Then, 1 mg of GP/PANI powder was dispersed in 1 mL of 2% APTES solution to prepare GP/PANI solution. Various amount of the GP/PANI solution (0, 4, 5, 6 and 7 µL) was drop casted onto electrode surface and dried overnight at room temperature in order to optimize the GP/PANI thickness on the electrode surface. After washing with ethanol, the electrode was then dried at 70 °C in the oven for 30 min. The CapAb was immobilized onto GP/PANI surface through a cross-linker EDC and NHS [27]. The electrode was then washed with PBS and immersed in 4 mL of 0.25% BSA solution for 1 h to avoid non-specific binding. Next, the modified electrode was incubated with 4 μL of *M. tuberculosis* CFP10 antigen solution for 1 h. After washing the electrode, 4 μL of the prepared Fe_3_O_4_/Au-Ab buffer solution was drop casted onto the surface and incubated for 40 min. After washing, the electrode was ready for measurement.

All CV measurements were carried out in 1 mM K_3_Fe(CN)_6_ with 50 mM KCl at potential range from −0.4 to 0.6 V for 40 cycles at scan rate of 100 mV/s. The DPV measurements were performed in 0.01 M phosphate buffered saline (PBS) with pH 7.4 at potential range from 0.0 to 1.0 V, step potential of 0.009 V and modulation amplitude of 0.0 V with the interval time of 0.64 s at room temperature.

## 3. Results and Discussion

### 3.1. Characterization of GP/PANI Nanocomposite Material

**Figure 2:**
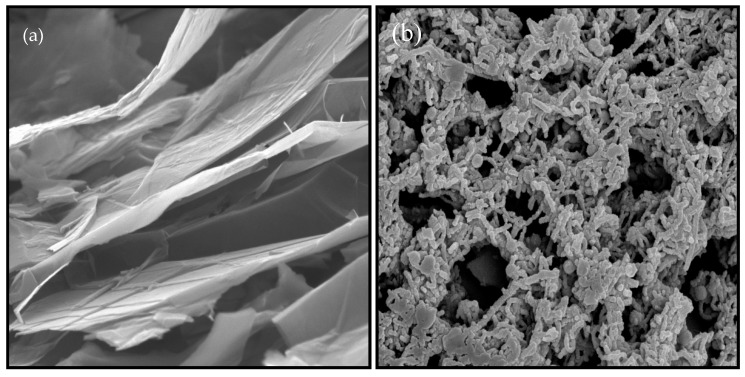

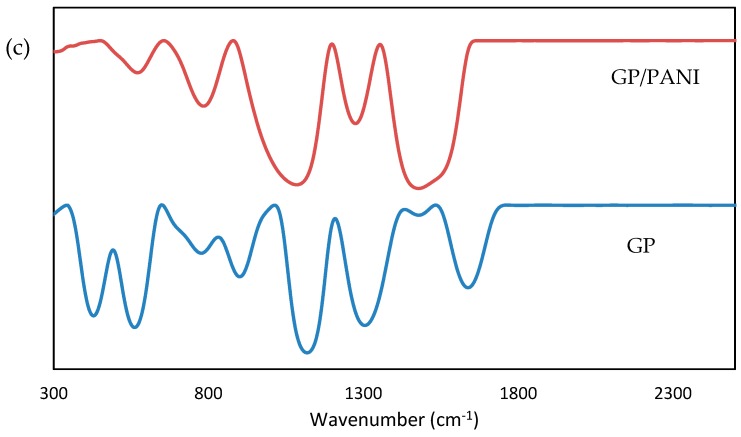
FESEM images of (**a**) graphene and (**b**) GP/PANI nanocomposite with magnification of 50k. (**c**) IR spectra of GP nanosheet and GP/PANI nanocomposite.

Figure 2 shows the scanning electron microscope (SEM) images of GP and GP/PANI nanocomposite. The SEM image of GP (Figure 2a) shows a good lamellar structure and rich wrinkled structures on the surface. Meanwhile, the SEM image of GP/PANI nanocomposite (Figure 2b) shows that the GP sheets are mostly covered with PANI nanofibers and formed cauliflower-like structures. It indicates that the presence of GP promotes the formation of agglomerate PANI nanofibers. Figure 2c presents IR spectra of GP nanosheet and GP/PANI nanocomposite. The peaks at 1655 cm^−1^ correspond to the C-C bond in GP. Compared to spectra of GP/PANI nanocomposite, the broad peaks at 1477 cm^−1^ indicates the N-H bond was overlapped with C=C aromatic. Besides that, the C-N peak appeared at 1274 cm^−1^ and 1084 cm^−1^. Therefore, we can conclude that the GP was successfully bound with PANI to form GP/PANI nanocomposite.

### 3.2. Characterization of GP/PANI-Modified SPGE

**Figure 3:**
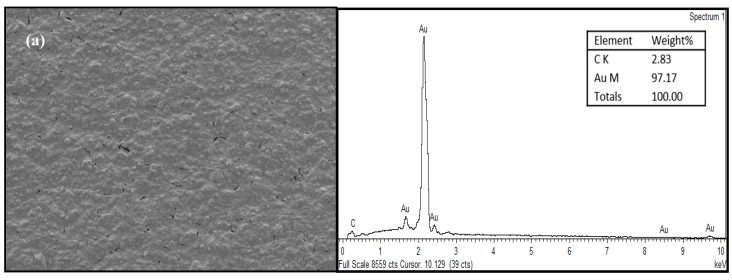

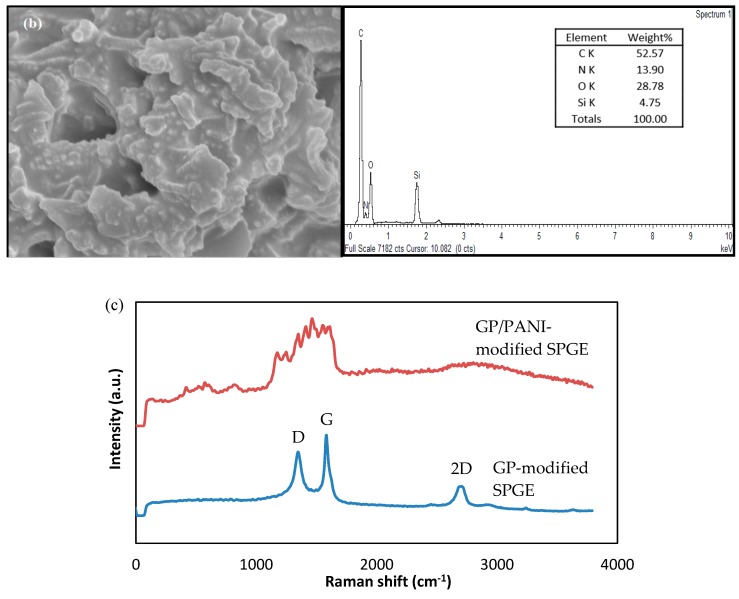
FESEM images coupled with EDX spectra of (**a**) bare SPGE and (**b**) GP/PANI-modified SPGE. (**c**) Raman spectra of GP-modified SPGE and GP/PANI-modified SPGE.

GP/PANI nanocomposite material was used to modify bare SPGE in order to enhance the sensor performance. The morphology of GP/PANI-modified SPGE surface was studied by FESEM-EDX. It was observed in Figure 3a,b that the surface structure of GP/PANI-modified electrode turned relatively rough and agglomerate compared to bare SPGE due to the formation of nanoparticle clusters on the electrode surface, which resulted in an increase of active surface area [28,29]. The presence of GP/PANI nanocomposite was also confirmed using EDX analysis. The presence of Si in GP/PANI-modified SPGE as depicted in Figure 3b was due to the APTES used as a dispersion agent. Meanwhile, the existence of N, O and a significant increase of C in GP/PANI-modified SPGE clearly indicated that the GP/PANI was successfully bound to the surface of SPGE. In addition, Raman spectroscopy was used to identify the presence of PANI in GP/PANI-modified electrode. As shown in Figure 3c, the spectrum of the GP-modified SPGE clearly shows three bands at 1350, 1580 and 2700 cm^−1^ which represent D, G and 2D bands, respectively. The G band represents the in-plane bond-stretching motion of the pairs of carbon sp^2^ atoms, while the D band is related to the conversion of a sp^2^-hybridized carbon to a sp^3^-hybridized carbon [18]. Compared with graphene, the D band of PANI showed a slight shift to higher frequencies, probably due to the strong long-range π-π and electrostatic interaction between PANI and GP-modified SPGE [30].

Next, the electrochemical behavior of GP/PANI-modified SPGE in potassium ferricyanide (K_3_Fe(CN)_6_) solution was investigated. K_3_Fe(CN)_6_ solution was used to study the electrocatalytic response of GP/PANI nanocomposite coated SPGE surface using the CV technique. It is notable that Ferrocyanide ion has been a good redox indicator for the analysis of the redox properties of modified electrodes due to its reversibility and rapid electrochemical reactions [31]. Moreover, it is believed that the thickness of GP/PANI nanocomposite on surface modification of SPGE played a crucial role for sensor performance. In this regard, we have studied different amounts of GP/PANI solution coated onto SPGE.

**Figure 4:**
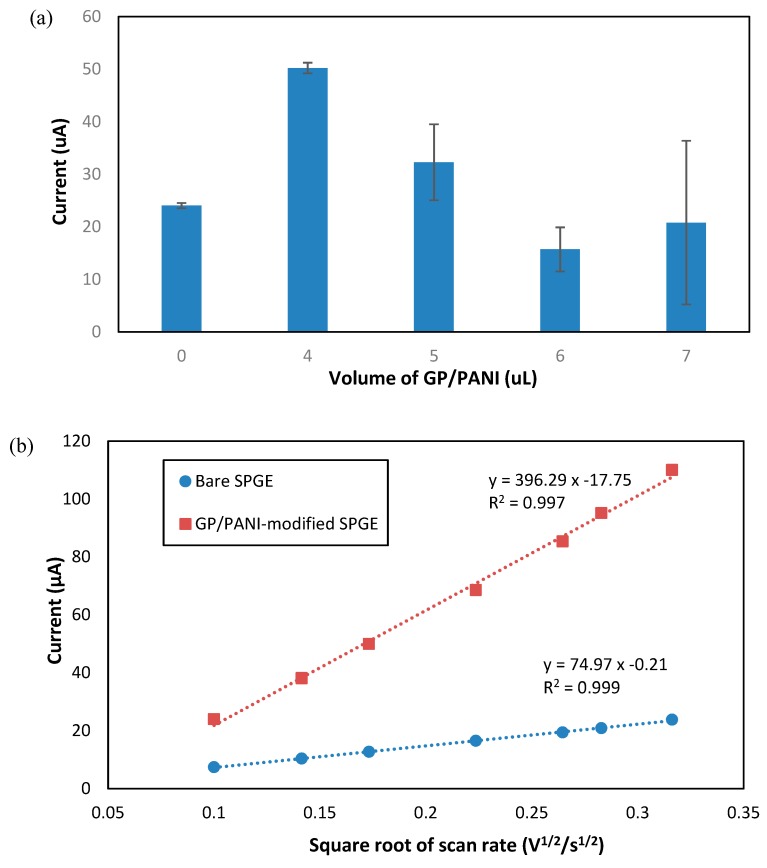
(**a**) Histogram of different amount of GP/PANI (µL) on SPGE. (**b**) The relationship of oxidation peak current and square root of scan rates for bare SPGE and GP/PANI-modified SPGE.

As depicted in Figure 4a, the sensor electrode with modification of 4 µL GP/PANI solution shows the highest current value approximately 50 µA. As the volume of GP/PANI solution was increased to 5 and 6 µL, the value of peak current decreased to 33 and 15 µA, respectively. However, it is noted that the peak current was slightly increased to 20 µA as the volume of GP/PANI solution increased to 7 µL. This could happen to the optimized value of 4 µL GP/PANI solution that gives highest surface electrostatic interactions, as compared to the GP/PANI volume solutions of 5, 6 and 7 µL. On the other hand, 4 µL of GP/PANI solution provides more stable of current observation (in terms of standard deviation) and higher peak current than bare SPGE. Thus, the thickness of GP/PANI particles on a SPGE electrode is crucial as it will affect the electrochemical performance. In this work, 4 µL GP/PANI solution was used to modify the SPGE surface.

To monitor the performance of the GP/PANI-modified SPGE for immunosensor development, the effective surface area for the electrode was studied and determined. To study the effective surface area of the electrode, the CV was performed in 1 mM K_3_Fe(CN)_6_ containing 50 mM KCl solution at different scan rates (10–100 mV/s). The effective surface area can be estimated according to the Randles-Sevcik equation [7].

(1) $$I_{p}=2.69\times 10^{5}\;A\times D^{1/2}n^{2/3}{\mathrm{Cv}}^{1/2},$$

where I_p_ represents the oxidation peak current (µA), n = 1 which is the number of electrons transferred, D is the diffusion coefficient of ferricyanide solution (7.6 × 10**^−^**^6^ cm^2^/s) [29], A is the electrode surface area (A) (cm^2^), C is the concentration of ferricyanide (mol/cm^3^) and v is the scan rate (V/s). Based on the graph of I_p_ versus v^1/2^ as shown in Figure 4b, the effective surface area values of bare SPGE and GP/PANI-modified SPGE were calculated as 0.101 and 0.535 cm^2^, respectively. The utilization of GP/PANI nanocomposite as electrochemical enhancer has led to five times greater sensor performance with respect to the bare SPGE. In addition, the linear relationship between redox peak current of GP/PANI-modified SPGE versus square root of scan rate (v^1/2^) reveals that the electrochemical reaction of the ferricyanide surface is a diffusion-controlled process [29].

### 3.3. Sandwich Electrochemical Immunosensor for CFP10 Detection Using GP/PANI-Modified SPGE

**Figure 5:**
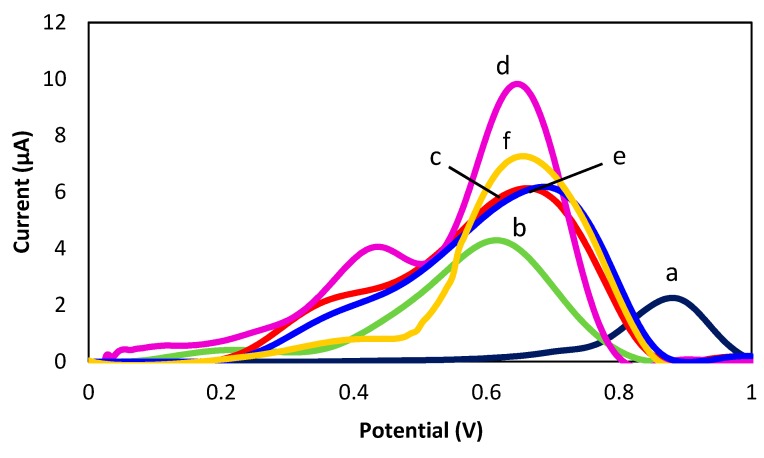
DPV responses of (**a**) bare SGPE; (**b**) SPGE/GP/PANI; (**c**) SPGE/GP/PANI/CapAb; (**d**) SPGE/GP/PANI/CapAb/CFP10/Ab-Fe@Au; (**e**) SPGE/GP/PANI/CapAb/BSA/Ab-Fe@Au and (**f**) SPGE/Fe@Au in 0.01 M PBS solution (pH 7.4).

A differential pulse voltammetry (DPV) technique was used in this study to measure the immunoreaction in detection of CFP10. Figure 5 shows the electrochemical responses of bare SGPE, SPGE/GP/PANI, SPGE/GP/PANI/CapAb, SPGE/GP/PANI/CapAb/CFP10/Ab-Fe@Au, SPGE/GP/PANI/CapAb/BSA/Ab-Fe@Au and SPGE/Fe@Au that were characterized using DPV in 0.01 M PBS (pH 7.4) containing 2.7 mM KCl. It is clearly observed that the bare SPGE (peak a) produce a small oxidation peak current compared to GP/PANI-modified SPGE (peak b). Besides that, the oxidation peak current of bare SPGE was observed at 0.9 V but after modification of electrode with GP/PANI, the oxidation peak current was shifted to a lower positive potential, which indicates that the developed immunosensor have improved the electronic transport capacity [13]. The enhanced electrochemical response in the oxidation processes indicated the electroconducting properties of the GP/PANI nanocomposite in facilitating the electron transfer between the electrode surface and chloride ion [13]. After stepwise immobilization the modified electrode with capture antibody (CapAb) (peak c) and Ab-Fe@Au with CFP10 antigen (peak d), the peak currents gradually increase might be due to the signal enhancement of Fe@Au MNPs [32]. Meanwhile, for the SPGE/GP/PANI/CapAb/BSA/Ab-Fe@Au (peak e), the peak current decrease, showing that the Fe@Au MNPs did not attach on the surface of the electrode.

**Figure 6:**
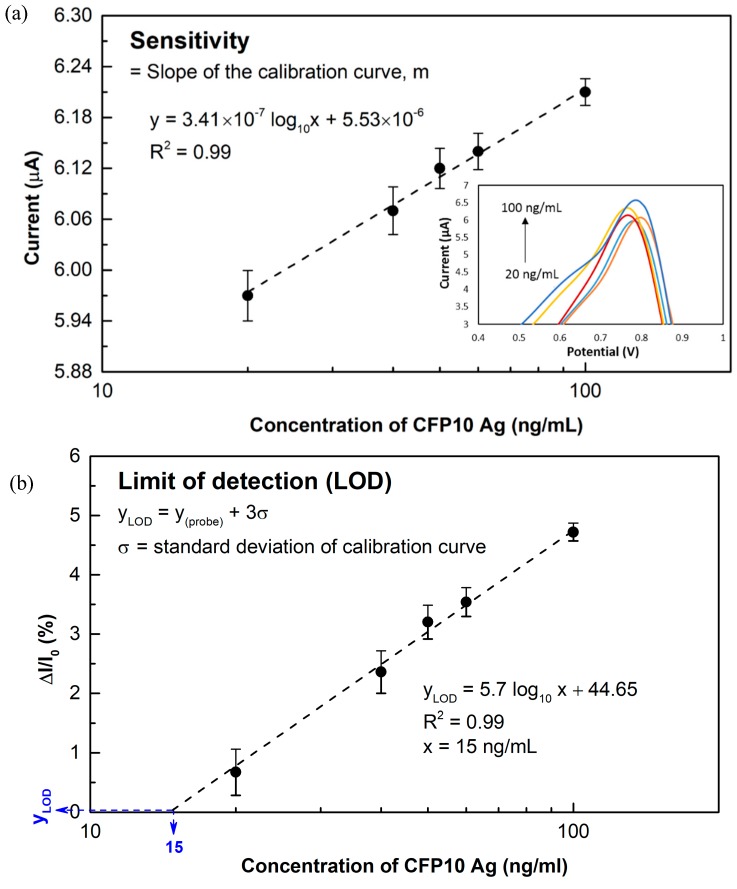
(**a**) Sensitivity study for the detection of CFP10 at concentrations in the range of 20–100 ng/mL. Inset image shows the DPV responses of the sensor towards the different concentrations of CFP10. (**b**) Calibration curve of the relative change in current with estimated LOD.

The sensitivity of DPV using the developed sensor towards different concentrations of *M. tuberculosis* CFP10 antigen was studied in the range of 20 to 100 ng/mL. The correlation between the DPV current and the target concentrations as obtained in Figure 6a indicates that the detection responses of the immunosensor was linear with the value of the complementary target with the linear regression of R^2^ = 0.99. An increase in peak current was observed as the concentration of CFP10 was increased. The device sensitivity was approximately 3.41 × 10^−7^ A/ng·mL^−1^ calculated from the curved plotted in the Figure 6a. The detection response of the developed immunosensor can be expressed as the relative change in current (ΔI/I_0_), where ΔI is the change in peak current after the target detection and I_0_ is the peak current corresponding to the probe (capture antibody) immobilization response [33]. The relative change of peak current towards different concentrations of CFP10 is plotted in Figure 6b. The significant current change around 5% was obtained as 100 ng/mL of CFP10 was employed on the developed immunoassay. As the concentration of CFP10 was decreased to 60, 50 and 40 ng/mL, a descending trend of detection response was observed approximately 3.5%, 3.2% and 2.4%, respectively. Meanwhile, merely 0.7% current change was recorded when 20 ng/mL of CFP10 was introduced. The limit of detection (LOD) can be defined as Y_LOD_ = Y_probe_ + 3σ; where Y_probe_ is the mean of the probe measurement and σ is the standard deviation of the calibration curve [34,35]. The linear regression equation of the plots shown in Figure 6b is Y_LOD_ = 5.7 log_10_ x + 44.65, where x is the concentration of target CFP10 antigen and Y_LOD_ is calculated from the equation. The calculated LOD obtained in this work was approximately 15 ng/mL. This was estimated as the lowest concentration of target CFP10 that can be provided a detectable electronic signal. The developed immunosensor assay can meet the demands of detecting the CFP10 in real sample, as the physiological range of interest of CFP10 is around 100 ng/mL [36]. Remarkably, this approach compared reasonably in term of linearity range and LOD with other previously reported CFP10-based detections as summarized in Table 1.

**Figure 7:**
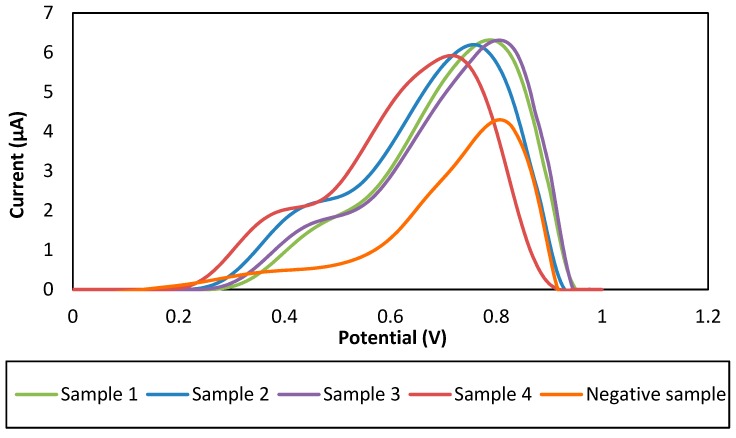
DPV response of sandwich electrochemical immunosensor for CFP10 detection using GP/PANI-modified SPGE in real sample application.

In order to test the validity of this method in real sample application, the developed immunosensor was then applied for the determination of *M. tuberculosis* CFP10 antigen in sputum samples. Five sputum samples consisted of four sputum samples from positive TB patients and one from a healthy person as the control experiment were collected from Hospital Universiti Sains Malaysia (HUSM). The fresh sputum samples were supplied to the Science laboratory at School of Medical Science (USM) and smear microscope examination was implemented prior to the culture method. The electrochemical analysis was performed simultaneously in the same workspace. We found that this electrochemical immunosensor showed the same trend of signals for all positive TB patients’ samples (Sample 1–Sample 4) as depicted in Figure 7, which obviously tended to give the highest signal change as compare to the healthy person sample (negative sample as control). From this viewpoint, we have verified that our developed GP/PANI modified SPGE electrode in a sandwich immunoassay format is feasible for early TB diagnosis through detection of the CFP10-specific antigen using sputum samples.

### 3.4. Reproducibility of the Developed Immunosensor

In biosensor performance assessment, reproducibility is a very significant feature in order to identify the reliability in working activity. It can be defined as the capability of a developed immunosensor of producing equivalent feedbacks for a repeated experimental setup [41]. Therefore, it is compulsory to test the reproducibility of the fabricated immunosensor for reliability verification of this method since it is a very significant parameter for immunosensor. For detection of 60 ng/mL CFP10, a series of five electrodes was setup. Peak currents within the range of 6.094–6.168 µA were measured from the developed immunosensors as tabulated in Table 2. Based on the measured peak currents for the five electrodes, the relative standard deviation (RSD) can be calculated from the following equation:

RSD = (σ/µ) × 100(2)

where σ is standard deviation and µ is the mean for the measurements. Acceptable RSD of 0.55% was obtained, which signify the developed immunosensor has an excellent reproducibility for the detection of low concentration CFP10 antigen.

## 4. Conclusions

In this study, we presented a novel detection strategy that uses GP/PANI-modified SPGE and sandwich immunoassay of TB marker protein CFP10 for early detection of TB infection. This platform employs the CapAb immobilized with GP/PANI as an electrochemical transducer and the Ab was conjugated by Fe_3_O_4_/Au MNPs allowing for the antigen-specific detection of probes from the assay solution. The morphological and surface analyses of GP/PANI modified on the SPGE surface were studied using FESEM equipped with EDX, which confirmed the existence of N, O and C elements of this nanocomposite on the surface electrode. The Raman spectra showed that the D band changes significantly with the presence of PANI in GP/PANI modified surface. The presence of the amine groups (NH_2_) in GP/PANI nanocomposite allowed the direct anchoring of the biolayer, which was identified by FTIR spectra at a peak of 1477 cm^−1^. It was observed that the integration of GP/PANI on SPGE represents an effective strategy in improving about five times sensor performance compared to unmodified SPGE. This method was capable to detect the CFP10 within 3 h with linearity in the range of 20–100 ng/mL (R^2^ = 0.99). The estimated LOD obtained in this work was 15 ng/mL. Moreover, the developed sandwich assay showed exceptional detection response towards CFP10 in sputum specimens for a real sample application and demonstrated high reproducibility (RSD of 0.55%) as a disposable-based immunoassay. Our findings signify that TB can be detected in a simple, sensitive and economic way through sputum-based observation, indicating that this assay analysis might be applied in the development of rapid-sensing tools for early TB monitoring.

## Figures and Tables

**Table 1:** Linearity range and LOD of CFP10 antigen using different immunoassay method.

No.	Detection Method	Linear Range	LOD	References
1.	Enzyme-linked immunosorbent assay (ELISA)	Not reported	0.35 IU/mL	[37]
2.	Plasmonic ELISA	0–0.1 µg/mL	0.01 µg/mL	[38]
3.	Magnetophoretic immunoassay	1 pM–1 mM	0.3 pM	[39]
4.	Surface plasmon resonance (SPR)	0.1 to 1 μg/mL	100 ng/mL	[40]
5.	Electrochemical	20–100 ng/mL	15 ng/mL	This work

**Table 2:** Reproducibility of 60 ng/mL of CFP10 antigen.

Replicate	Peak Current (µA)	Mean, µ	Standard Deviation, σ	Relative Standard Deviation, RSD (%)
1	6.09	6.13	0.034	0.55
2	6.16			
3	6.16			
4	6.14			
5	6.10			
